# Supplementary material for: Treatment response in dogs with naturally occurring grade 3 elbow osteoarthritis following intra-articular injection of 117mSn (tin) colloid
Source: PLoS One. 2021 Jul 19;16(7):e0254613. doi: 10.1371/journal.pone.0254613 (PMC8289027; doi:10.1371/journal.pone.0254613)
Supplement: Attachment — Submitted filename: Response to Reviewers (PONE-D-21-08558) 6.10.21.docx [file pone.0254613.s001.docx]

**Response to Reviewers (manuscript PONE-D-21-08558, Donecker et al)**

***Academic Editor’s comment (Dr. Carreira):***  Provide a brief description of the anesthetic protocol.

***Authors’ response:*** The anesthetic protocol is described in the revised manuscript (Materials and Methods, Therapeutic Device and Administration Procedure).

***Academic Editor’s comment:*** The therapeutics that each patient used in the study is missing.

***Authors’ response:*** A table (Table 1) has been added to the manuscript (Materials and Methods, Study Animals) listing the various therapeutic agents for joint pain used by dogs enrolled in the study.

***Academic Editor’s comment:***  Patient evolution should be provided with radiographs made during the study or at beginning and end.

***Author’s response:*** It would be impractical for the manuscript to include intra-study radiographs of all 14 per-protocol dogs. Instead, we have included high-quality pre- and post-treatment radiographs of a representative dog enrolled in the study with grade 3 elbow osteoarthritis that exhibited a conservative but favorable response to therapy. The radiographs are arranged as a four-part, multi-panel figure submitted as a TIFF file (Figure 1).

***Academic Editor’s comment:***  P-values should be presented as two-decimal values.

***Authors’ response:*** P-values are expressed as two-decimal values in the revised manuscript.

***Revised manuscript cover letter:*** As requested in the Editorial Manager’s e-mail message of April 28, 2021, the authors are providing:

- An amended Competing Interests Statement that explicitly identifies Exubrion Therapeutics as a commercial funder of the study.
- Confirmation that we adhere to all PLOS ONE policies on data and materials sharing. All supporting references cited in the manuscript are published and available in the public domain.
- A statement that there are no ethical or legal restrictions on data sharing.

***Review comments by Reviewer #1:*** This is an uncontrolled longitudinal study of pain reduction with a secondary analysis of agreement of assessment methods. I have some concerns about the design and statistical aspects of the study. While longitudinal, the data are not analyzed longitudinally. Instead, a p-value is calculated at each time point. Presumably p<0.05 is an indication that of success. However, p-values have little meaning in the context of multiple testing, and it is not clear what is the specific hypothesis being tested to determine the specific aim of the study. The authors rightly point out that they are unable to compare to a control, so this is largely just an exploratory analysis at each time point with p-values being calculated, with no concern about type I error control. Attendant to that, there is no statistical justification of sample size or discussion of power or type I error preservation, likely because it is not clear what specifically is being tested, except using p-values as a guide to see if there is pain reduction. Similarly, McNemar's test is used to determine agreement, where agreement is based on a p-value > 0.05. Since a nonsignificant p-value does not prove the null hypothesis, it again serves only as a guide or experimental metric to indicate that there does not appear to be significant disagreement in the assessments at alpha=0.05. The paper would be have been strengthened by determining a criterion for improvement based on a hypothesis test, which could be a significant rate of change parameter from a repeated measures regression model with property controlled type I error rate. Under that hypothesis, one could then see if there is sufficient power to draw conclusions based on the sample of dogs selected.

***Authors’ response:*** Time is considered as a fixed main effect with ordering of the levels so that the time points can be compared in a meaningful way, and not just “all vs. all.” Regarding the power of the McNemar’s test, with such a small sample, there is essentially no power to discriminate, and results are an indicator of the advisability of more research using a much larger study sample. The description of the statistical analysis in the manuscript has been revised to include additional detail in view of the reviewer’s comments.

***Review comments by Reviewer #2:*** Where are the radiographic images of affected joints before and after treatment at different intervals?

***Authors’ response:*** See response to the Academic Editor’s comment above. Radiographs of a representative case (Figure 1) are included in the manuscript, along with corresponding CBPI and clinician evaluation data for this patient (Table 5).
